# BOP1 Used as a Novel Prognostic Marker and Correlated with Tumor Microenvironment in Pan-Cancer

**DOI:** 10.1155/2021/3603030

**Published:** 2021-09-22

**Authors:** Wei Li, Peipei Song, Mengyuan Zhao, Lina Gao, Jianqin Xie, Chongge You

**Affiliations:** ^1^Laboratory Medicine Center, Lanzhou University Second Hospital, Lanzhou 730030, China; ^2^Department of Geriatrics, Xiangya Hospital, Central South University, Changsha 410008, China

## Abstract

Previous studies have indicated the important role of block of proliferation 1 (BOP1) in the progression of several malignant tumors; no comprehensive pan-cancer analysis of BOP1 has been performed. Here, we aim to systematically identify the expression, prognostic value, and potential immunological functions of BOP1 in 33 malignancies. We obtained the gene expression data and clinical information from multiple public databases to assess the expression level and prognostic value of BOP1 in 33 cancers. We also analyzed the relationship between BOP1 expression and DNA methylation, tumor microenvironment (TME), microsatellite instability (MSI), tumor mutational burden (TMB), and immune checkpoints. Moreover, we conducted gene set enrichment analysis (GSEA) to investigate the biological function and signal transduction pathways of BOP1 in different types of tumors. Finally, we validated the expression of BOP1 in lung cancer cell line and detected the influence of BOP1 on lung cancer cell migration and the expression of epithelial-mesenchymal transition- (EMT-) related genes. Collectively, our findings elucidated that BOP1 has the potential to be a promising molecular prognostic biomarker for predicting poor survival in various malignant tumors, as well as a cancer-promoting gene involved in tumorigenesis and tumor immunity.

## 1. Introduction

Cancer has been one of the main causes of deaths of the population worldwide [1]. The latest cancer statistic data indicate that there were 19.3 million new cases and more than 10.0 million deaths worldwide in 2020 [2]. Although a variety of treatments, such as chemotherapy, surgery, radiotherapy, and immunotherapy, have been used to treat cancer patients in recent decades, the prognosis for cancer patients remains unfavorable, particularly in advanced tumor patients. As a result, further research into the molecular pathogenesis underlying tumors is urgently needed in order to develop effective diagnostics and therapies. High-throughput, large size, multi-omics, and multitumors data will contribute to identify the key factors of tumorigenesis. Many public databases, such as Gene Expression Omnibus (GEO) and The Cancer Genome Atlas (TCGA), have been continuously developed and improved in recent years, containing a significant amount of multi-omics data of tumors that allow for pan-cancer analysis [3–5].

Block of proliferation 1 (BOP1) is a conserved RNA-binding protein involved in ribosome biogenesis, cell cycle, and cell proliferation [6]. A recent study showed that knockdown of BOP1 attenuates vascular smooth muscle cell proliferation and migration via activating the p53-dependent pathway [7]. Moreover, it is reported that BOP1 is dysregulated in several cancers and is involved in promoting the tumor occurrence and progression [8–11]. BOP1 is expressed at higher expression levels in colorectal cancer, which is associated with a poor patient prognosis, while BOP1 silencing mitigates tumor cell proliferation, migration, and invasion and enhances cell apoptosis [10–12]. Similar results have been found in gastric cancer [8], prostate cancer [9], and hepatocellular carcinoma (HCC) [13]. Nevertheless, it is uncertain if BOP1 plays crucial roles in other cancers, especially lung cancer. To date, there are no comprehensive studies on the clinical significance and potential biological function of BOP1 in pan-cancer.

In the present study, we obtained the gene expression data and clinical information from several public databases to assess the expression level and prognostic value of BOP1 in 33 types of cancer. We investigated the relation between BOP1 expression and DNA methylation, tumor microenvironment (TME), microsatellite instability (MSI), tumor mutational burden (TMB), and immune checkpoints. Meanwhile, we conducted gene set enrichment analysis (GSEA) to investigate the biological function and signal transduction pathways of BOP1 in different types of tumors. We also used reverse transcription-quantitative polymerase chain reaction (RT-qPCR) to confirm BOP1 expression in lung cancer cell lines. Furthermore, we detected the influence of BOP1 on lung cancer cell migration and epithelial-mesenchymal transition- (EMT-) related genes' expression. Our results indicate that BOP1 is associated with tumor microenvironment and might be used as a novel prognostic factor in pan-cancer.

## 2. Materials and Methods

### 2.1. Data Acquisition and Expression Analysis

The gene expression profiles, somatic mutation, and related clinical data in TCGA, Genotype-Tissue Expression (GTEx), and Cancer Cell Line Encyclopedia (CCLE) were downloaded from the UCSC XENA website (https://xenabrowser.net/datapages/). The expression of BOP1 was analyzed in 31 normal tissues, 33 cancer tissues, and 21 tumor cell lines. All data analyses were performed by utilizing R (V.4.0.5, https://www.r-project.org/), and the R packages “ggradar” and “ggplot2” were used to draw radar graphs and box plots, respectively. The protein expression data of BOP1were acquired from the UALCAN databases (http://ualcan.path.uab.edu/) and were compared to the immunohistochemistry (IHC) staining results from the Human Protein Atlas (HPA) database (http://www.proteinatlas.org/).

### 2.2. Survival Analysis of BOP1 in Pan-Cancer

The survival data and BOP1 expression value of each sample were extracted from TCGA data sets. According to the median expression levels of BOP1, patients were divided into high-risk groups and low-risk groups. The univariate Cox analysis performed by R packages “survival” and “forestplot” was used to investigate the prognostic value of BOP1 expression regarding overall survival (OS), disease-specific survival (DSS), disease-free interval (DFI), and progression-free interval (PFI). Besides, the Kaplan–Meier curves were constructed using the R packages “survival” and “survminer” to reveal the differences in patient survival of each cancer.

### 2.3. Genetic Alteration and Methylation Modification Analysis

The copy-number alterations and mutations analysis of BOP1 based on TCGA data were implemented by cBioPortal (https://www.cbioportal.org/). The HM450 methylation data of each tumor were also derived from the cBioPortal database. The connection between the BOP1 expression levels and methylation levels in its promoter region was analyzed for each cancer and visualized using the R package “ggpubr.” The correlation between BOP1 methylation and prognosis, including OS, PFI, DFI, and DSS, was also investigated using Kaplan–Meier survival analysis.

### 2.4. Gene Set Enrichment Analysis of BOP1 in Cancers

The GSEA was performed by using R package “clusterProfiler” to investigate the biological function and potential signaling pathway of BOP1 in each cancer. Kyoto Encyclopedia of Genes and Genomes (KEGG) and Gene Ontology (GO) gene data were obtained from the GSEA online server (https://www.gsea-msigdb.org/gsea/index.jsp).

### 2.5. Correlation Analysis between BOP1 Expression and Tumor Microenvironment

The tumor microenvironment characterizations in 33 types of tumor were identified by using the method reported by Zeng et al. [14]. For each cancer specimen, the TMEscore (related to TME infiltration), TMEscoreA (related to immune-relevant signatures), and TMEscoreB (related to stromal-relevant signatures) were calculated. A heat map was generated using the R packages to summarize the association of BOP1 expression with immune-relevant signatures, stromal-relevant signatures, and mismatch DNA repair signatures. Besides, the immune cell infiltration data were downloaded from the ImmuCellAI database (http://bioinfo.life.hust.edu.cn/web/ImmuCellAI/). The same method was exploited to analyze the association between BOP1 expression and the level of immune cell infiltration.

### 2.6. Correlation of BOP1 Expression with Immune Checkpoints, MSI, and TMB

The somatic mutation data of 33 tumors were downloaded from GDC TCGA cohort in UCSC XENA. The TMB score was calculated by dividing the quantities of somatic mutations by the total length of the exons. The MSI data were obtained from a recent study [15]. The connections between BOP1 expression and MSI values and TMB values were evaluated by utilizing Spearman's correlation coefficient. Moreover, the relationship between BOP1 and the immune checkpoints PDCD1, KLRB1, CTLA4, LAG3, and TIGIT was analyzed based on TCGA data.

### 2.7. Cell Culture

The lung carcinoma cell line A549 was purchased from HonorGene (Changsha, China). A549 cells stably transfected with BOP1-shRNA or pCDH-BOP1 or negative control vector were previously constructed in our laboratory. All cells were cultured in DMEM containing 10% fetal bovine serum and incubated at 37°C in humidified air with 5% CO_2_.

### 2.8. Transwell Assay

In total, 100 ul of cell suspension containing 1 × 10^4^ cells was added to the upper Transwell chamber (Corning, USA), and the complete medium containing 10% FBS was seeded into to the basal chamber. After incubation for 48 h, the cells in the upper chamber were removed. Then, all cells in the lower chamber were fixed in a mixture of methanol and acetone for 20 minutes and stained with crystal violet (0.5%; Solarbio, China) for 5 minutes. With a light microscope, at least 5 fields of view were randomly selected for each group to take pictures and count the cells.

### 2.9. RNA Extraction and RT-qPCR

According to product instructions, the TRIzol reagent (Thermo Fisher Scientific, MA, USA) was used to isolate total RNA from cells. cDNA was reverse-transcribed from total RNA using HiFiScript cDNA Synthesis Kit (Cwbiotech, Beijing, China). RT-qPCR was conducted using PikoReal Real-Time PCR System (Thermo Fisher Scientific, MA, USA). The primer sequences of BOP1 were 5′-GTGGTACGATGACTTCCCCC-3′ (forward primer) and 5′-GAAGCCCACATCCCCAAACT-3′ (reverse primer). GAPDH was used as an internal control.

### 2.10. Western Blotting

All the cells were incubated with RIPA buffer (Beyotime, Shanghai, China). The bicinchoninic acid (BCA) protein assay kit (Beyotime, Shanghai, China) was used to detect the total protein concentration for each sample. Proteins were separated by SDS-PAGE electrophoresis. After electrophoresis, the protein was transferred to a nitrocellulose (NC) membrane and then blocked with 5% skim milk. Next, the membranes were incubated with the following primary antibodies: anti-E-cadherin antibody (#60335-1-Ig, 1:4000; Proteintech, USA), anti-N-cadherin antibody (#22018-1-AP, 1:3000; Proteintech, USA), and anti-Snail antibody (#13099-1-AP, 1:750; Proteintech, USA). Subsequently, the membranes were incubated with corresponding secondary antibodies conjugated to horseradish peroxidase (HRP). An enhanced chemiluminescence system was used to visualize immunoreactive proteins on the membranes.

### 2.11. Statistical Analysis

The R software (version 4.0.5) and GraphPad prism software (version 8.0) were exploited to execute the corresponding statistical analyses in this study. The *T*-test or Mann–Whitney test was used to compare the difference between two groups. Pearson's test or Spearman's test was used to assess the correlation of two variables. The survival analysis was implemented by using the Cox proportional hazard regression analysis and Kaplan–Meier curve with a log-rank test. Statistical significance was defined as a *P* value of less than 0.05.

## 3. Results

### 3.1. Expression of BOP1 in Pan-Cancer and Normal Tissue Samples

**Figure 1:**
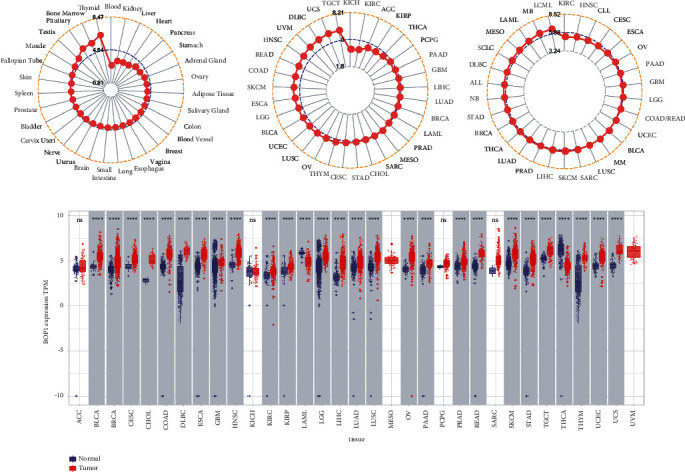
The differential expression of BOP1 mRNA in pan-cancer. (a) Mean expression of BOP1 in normal tissues. (b) Mean expression of BOP1 in TCGA. (c) Mean expression of BOP1 in tumor cell lines. (d) Expression of BOP1 between cancer tissues and corresponding normal tissues (^*∗∗∗∗*^*P* < 0.0001; NS, no statistical difference).

**Figure 2:**
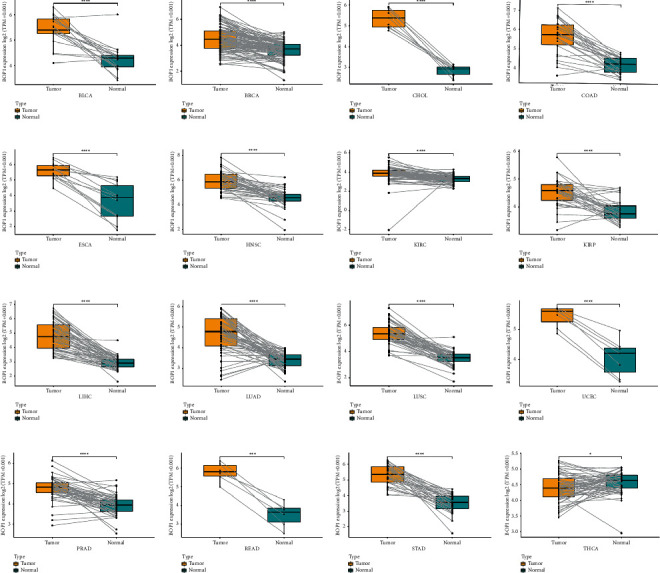
BOP1 expression in paired tumor samples and adjacent normal tissues in 16 cancers (^*∗*^*P* < 0.001, ^*∗∗∗*^*P* < 0.0001, and ^*∗∗∗∗*^*P* < 0.0001).

We analyzed the expression data of BOP1 in the GTEx database, and the results showed that BOP1 was expressed in various normal tissues, with the highest expression level in the thyroid and the lowest in the blood sample (Figure 1(a)). Then, we investigated the expression of BOP1 in 33 cancer samples from TCGA database and ranked them according to the mean value from low to high. BOP1 is expressed in all tumor tissues, as shown in Figure 1(b), with the highest level in testicular germ cell tumors (TGCT) and the lowest level in the kidney chromophobe (KICH). We also evaluated the level of BOP1 in different cancer cells from the CCLE database. Our results suggest that the expression level of BOP1 is highest in chronic myelogenous leukemia (LCML), while it is lower in most other tumor types (Figure 1(c)). Next, we used TCGA and GTEx data to compare the expression of BOP1 between cancer tissues and corresponding normal tissues in 33 cancers. We found that BOP1 was dysregulated in 27 cancer tissues (Figure 1(d)). Among them, BOP1 was upregulated in bladder urothelial carcinoma (BLCA), cervical squamous cell carcinoma and endocervical adenocarcinoma (CESC), colon adenocarcinoma (COAD), breast invasive carcinoma(BRCA), cholangiocarcinoma (CHOL), glioblastoma multiforme (GBM), esophageal carcinoma (ESCA), lymphoid neoplasm diffuse large B-cell lymphoma (DLBC), head and neck squamous cell carcinoma (HNSC), kidney renal papillary cell carcinoma (KIRP), brain low-grade glioma (LGG), kidney renal clear cell carcinoma (KIRC), lung squamous cell carcinoma (LUSC), lung adenocarcinoma (LUAD), liver hepatocellular carcinoma (LIHC), prostate adenocarcinoma (PRAD), ovarian serous cystadenocarcinoma (OV), testicular germ cell tumors (TGCT), pancreatic adenocarcinoma (PAAD), rectum adenocarcinoma esophageal carcinoma (READ), stomach adenocarcinoma (STAD), skin cutaneous melanoma (SKCM), thymoma (THYM), uterine carcinosarcoma (UCS), and uterine corpus endometrial carcinoma (UCEC) (*P* < 0.05). However, compared with normal tissues, the levels of BOP1 were decreased in acute myeloid leukemia (LAML) and thyroid carcinoma (THCA) (*P* < 0.05). There was no significant difference in the expression of BOP1 in adrenocortical carcinoma (ACC), KICH, pheochromocytoma and paraganglioma (PCPG), and sarcoma (SARC) (*P* > 0.05). Consistently, BOP1 was consistently upregulated in paired tumor samples compared with adjacent normal samples in 16 malignancies, including BRCA, BLCA, COAD, CHOL, HNSC, ESCA, LIHC, KIRC, KIRP, LUSC, LUAD, UCEC, THCA, STAD, READ, and PRAD (*P* < 0.05) (Figure 2).

**Figure 3:**
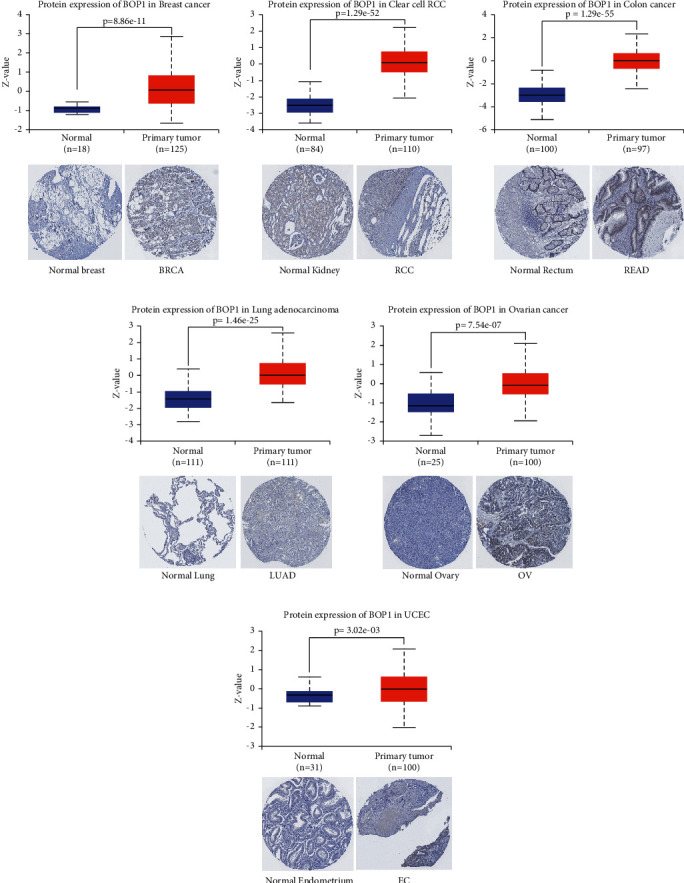
The expression of BOP1 protein in 6 tumors.

In addition, we investigated the protein expression data of BOP1 in the UALCAN databases and compared it to the IHC staining results from the HPA database. The results indicated that the expression of BOP1 protein in BRCA, clear cell renal cell carcinoma (ccRCC), COAD, LUAD, OV, and UCEC was significantly higher than that in corresponding normal tissues (*P* < 0.05) (Figure 3), consistent with the results of BOP1 mRNA expression levels from TCGA. The IHC staining results also showed that normal breast, lung, ovary, rectum, and endometrium tissues with weak or no BOP1 IHC staining intensity, while corresponding tumor tissues with more stronger staining intensity (Figure 3). IHC staining intensity was modest in both normal kidney tissue and ccRCC tumor tissue (Figure 3(b)).

### 3.2. Prognostic Value of BOP1 in Various Cancers

**Figure 4:**
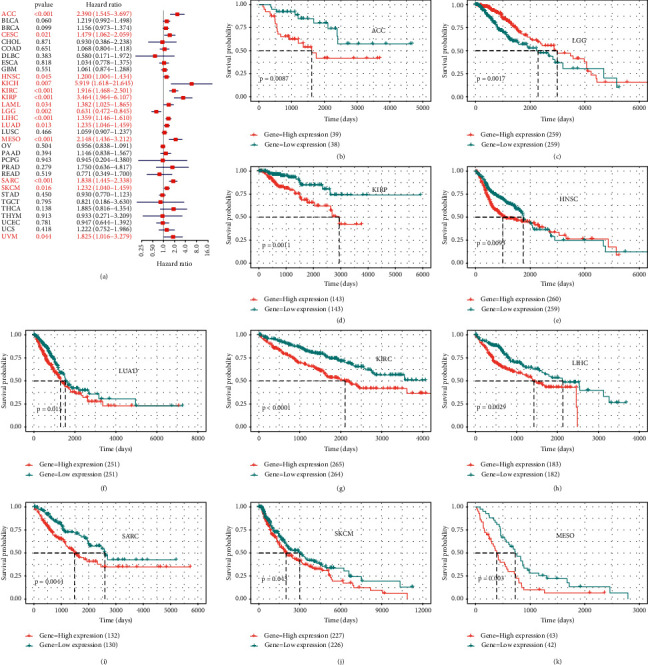
The correlation of BOP1 expression and OS in 33 cancers. (a) Forest plot of OS based on univariate Cox regression in 33 tumors. (b–k) Kaplan–Meier curves of OS for BOP1 expression.

**Figure 5:**
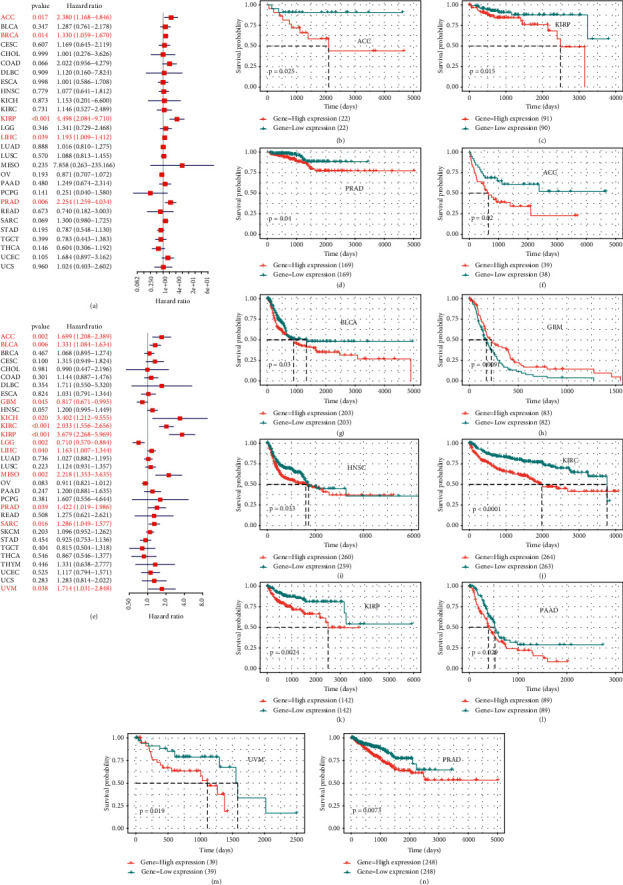
The correlation of BOP1 expression and DFI and PFI in 33 cancers. (a) Forest plot of DFI in 33 tumors. (b–d) Kaplan–Meier curves of DFI for BOP1 expression. (e) Forest plot of PFI in 33 tumors. (f–n) Kaplan–Meier curves of PFI for BOP1 expression.

**Figure 6:**
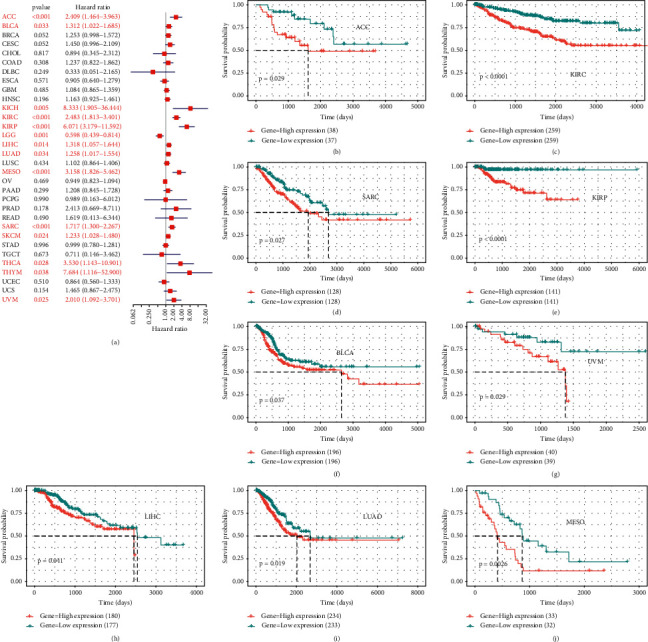
The correlation of BOP1 expression and DSS in 33 cancers. (a) Forest plot of DSS based on univariate Cox regression in 33 tumors. (b–j) Kaplan–Meier curves of DSS for BOP1 expression.

To investigate the prognostic value of BOP1 in human cancers, we used RNA-sequencing data from TCGA to implement a survival analysis for each tumor. In 14 malignancies, including ACC, CESC, KIRC, KICH, HNSC, KIRP, LAML, SKCM, SARC, MESO, LUAD, LIHC, LGG, and UVM, the expression of BOP1 was shown to be associated with OS (*P* < 0.05). Of these, the BOP1 expression level was one of the protective elements in LGG, and it was a high-risk factor for the other 13 cancers (Figure 4(a)). According to the Kaplan–Meier survival analysis, high BOP1 expression was similarly linked to a poor OS in individuals with ACC (*P* < 0.01), KIRP (*P* < 0.01), HNSC (*P* < 0.01), KIRC (*P* < 0.001), LIHC (*P* < 0.01), LUAD (*P* < 0.05), MESO (*P* < 0.01), SARC (*P* < 0.01), or SKCM (*P* < 0.05), while low BOP1 expression was related to poor OS in LGG (*P* < 0.01) (Figures 4(b)–4(k)). With regard to DFI, the high expression of BOP1 was a risk factor in BRCA, PRAD, LIHC, ACC, and KIRP (Figure 5(a)). The Kaplan–Meier survival curve also revealed that the BOP1 expression level was significantly correlated with poor prognosis in ACC (*P*=0.025), KIRP (*P*=0.015), and PRAD (*P*=0.04) (Figures 5(b)–5(d)). Moreover, high BOP1 expression was revealed to be a risk factor in ACC, BLCA, KICH, KIRC, KIRP, LIHC, MESO, PRAD, SARC, and UVM, whereas it was a protective factor in GBM and LGG (Figure 5(e)). Similar results were observed in the Kaplan–Meier analysis. It was discovered that a high BOP1 level was related to poor PFI in BLCA, ACC, HNSC, KIRP, PAAD, UVM, KIRC, and PRAD, whereas a low BOP1 level was linked to poor PFI in GBM patients (Figures 5(f)–5(n)). Eventually, taking into account the odds of death from non-neoplastic causes during the follow-up period, we evaluated the connection between BOP1 expression levels and DSS in 33 cancers. The forest plots showed that BOP1 was a protective gene in LGG but a high-risk gene in ACC, BLCA, SKCM, SARC, KIRP, KIRC, KICH, LUAD, LIHC, THCA, THYM, UVM, and MESO (Figure 6(a)). In addition, the survival analysis indicated that BOP1 expression was positively related to DSS in ACC, KIRC, KIRP, BLCA, LIHC, LUAD, MESO, SARC, and UVM (Figures 6(b)–6(j)).

### 3.3. Genetic Alteration and Methylation Modification of BOP1 in Pan-Cancer

**Figure 7:**
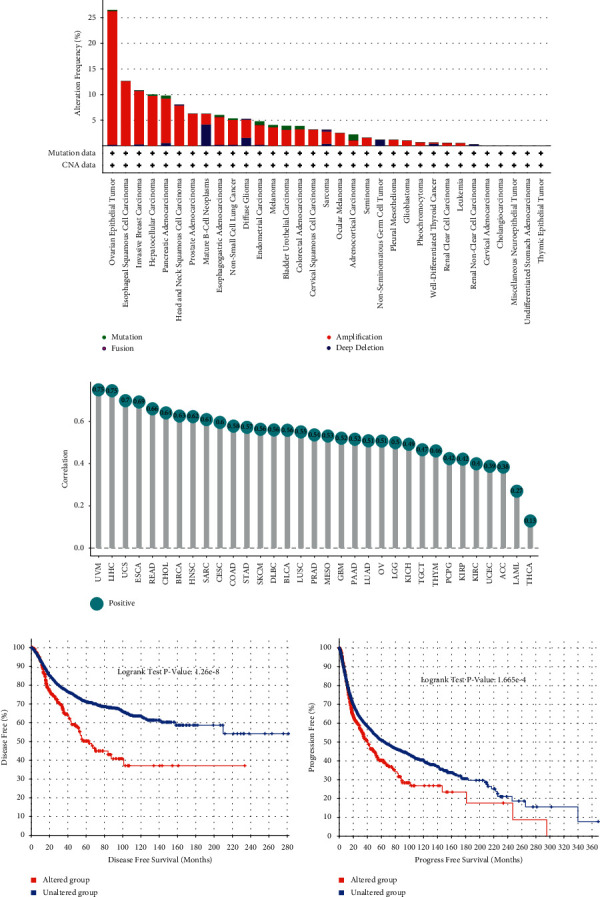
Genetic alteration of BOP1 in pan-cancer.

The copy-number alterations and mutations analysis of BOP1 were conducted based on TCGA data by using cBioPortal. We found that BOP1 was altered in 28 cancers, with amplification being the most frequent alteration (Figure 7(a)). Then, we further analyzed the correlation between the copy number of BOP1 and its expression level in 33 cancers. BOP1 expression levels were significantly positively correlated with its gene copy-number (*P* < 0.01), as shown in Figure 7(b), notably in UVM, LIHC, UCS, ESCA, and READ. Given that alterations in BOP1 affecting its expression level, we explored the relationship between BOP1 alterations and prognosis of tumor patients. Our results suggested that patients with BOP1 alterations had shorter progression-free survival (PFS) and disease-free survival (DFS) than those who without BOP1 alterations (*P* < 0.001) (Figures 7(c) and 7(d)).

**Figure 8:**
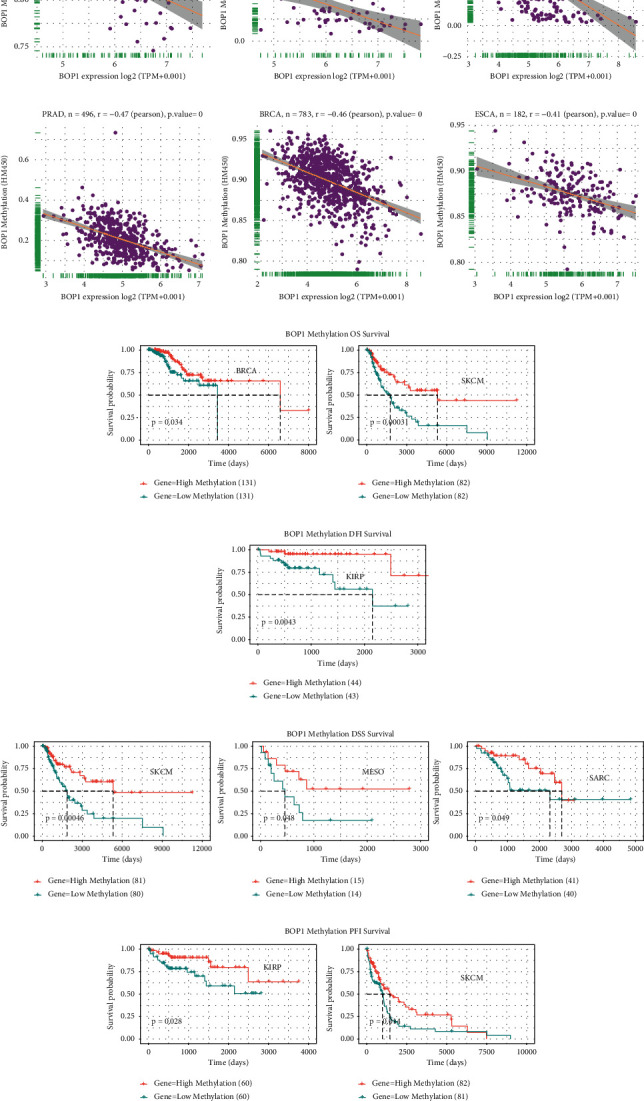
Methylation modification of BOP1 in pan-cancer. (a) The relevance between BOP1 level and promoter methylation in UVM, UCS, SARC, PRAD, BRCA, and ESCA. (b) The relevance between BOP1 promoter methylation level and OS in BRCA and SKCM. (c) The relevance between BOP1 promoter methylation level and DFI in KIRP. (d) The relevance between BOP1 promoter methylation level and DSS in SKCM, MESO, and SARC. (e) The relevance between BOP1 promoter methylation level and PFI in KIRP and SKCM.

Considering methylation modification was an important means of regulating gene expression, we used the cBioPortal data to investigate the relationship between the methylation levels of BOP1 gene promoter and BOP1 expression levels. BOP1 gene promoter methylation was shown to be substantially associated with BOP1 expression in 17 tumors, with 16 negative correlations and 1 positive correlation (Supplementary Table S1). The six strongest correlations (UVM, UCS, SARC, PRAD, BRCA, and ESCA) are shown in Figure 8(a). Subsequently, the survival analysis was performed to investigate the connection between the BOP1 promoter region methylation level and patient prognosis. The Kaplan–Meier curve revealed that the BOP1 promoter methylation level was positively related to OS in BRCA and SKCM (Figure 8(b)), while a low degree of BOP1 promoter methylation was associated with poor DFI only in patients with KIRP (Figure 8(c)). For DSS, the BOP1 promoter methylation level was only a protective factor in SKCM, MESO, and SARC (Figure 8(d)). Furthermore, in KIRP and SKCM, PFI was lower in patients with a low BOP1 promoter methylation level than in those with a high methylation level (Figure 8(d)).

### 3.4. Gene Set Enrichment Analysis of BOP1 in Cancers

**Figure 9:**
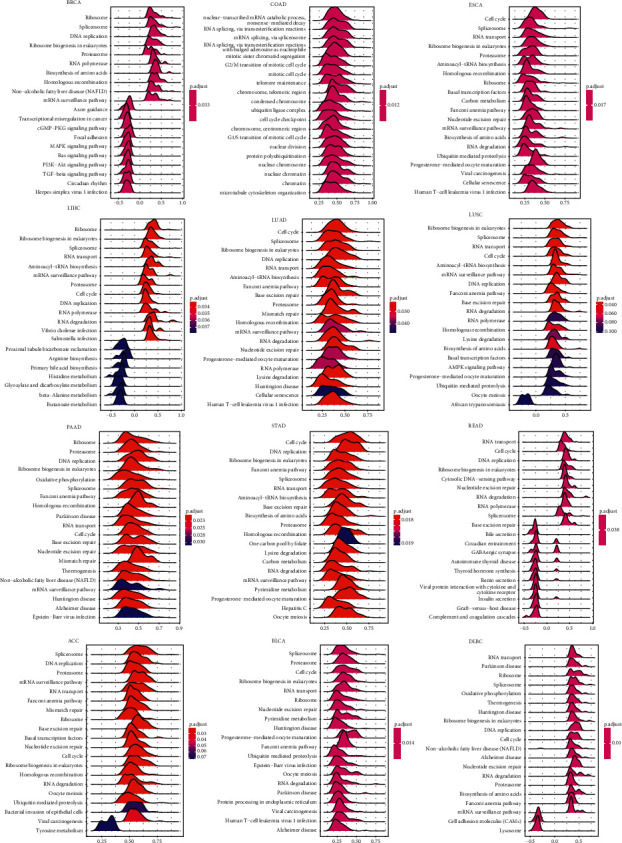
GSEA of BOP1 in 12 types of tumors.

To explore the potential functions of BOP1 in cancers, we conducted a GSEA to analyze which KEGG pathways were associated with BOP1 expression in each tumor. It was found that the expression of BOP1 was related to multiple tumorigenesis and immune regulation-related pathways in various cancers, such as the cell cycle, RNA transport, RNA degradation, RNA splicing, DNA repair, DNA replication, spliceosome, ribosome, mRNA surveillance pathway, viral carcinogenesis, ribosome biogenesis in eukaryotes, biogenesis of amino acids, and proteasomes (Figure 9).

### 3.5. Correlation Analysis between BOP1 Expression and Tumor Microenvironment

**Figure 10:**
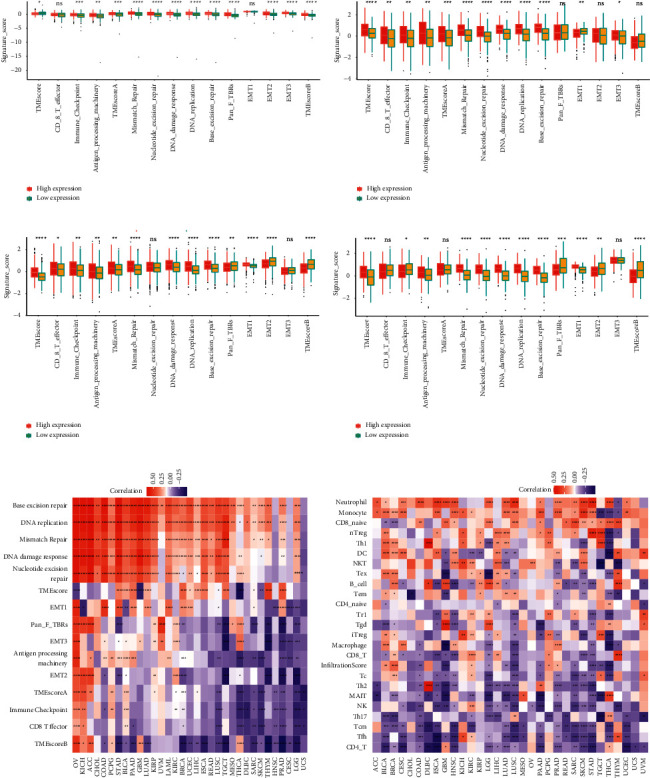
Correlation analysis between BOP1 and tumor microenvironment: (a) OV, (b) BLCA, (c) BRCA, and (d) STAD. (e) Correlation between BOP1 expression and TME-related biological processes in 33 tumors. (f) Correlation between BOP1 expression and immune cell infiltration. ^*∗*^*P* < 0.05, ^*∗∗*^*P* < 0.01, and ^*∗∗∗*^*P* < 0.001.

Tumor microenvironment plays key roles in the occurrence, development, and metastasis of cancers. We calculated the TMEscore and differentially expressed TME signatures according to BOP1 expression in 33 types of tumor, and we investigated the correlation between BOP1 expression and TMEscores, and relevant biological processes were determined in each tumor. We found that many differentially expressed TME signature genes between samples with BOP1 high and low expression, including genes involved in the immune regulation, DNA damage repair, and EMT (Figures 10(a)–10(d)). The association between BOP1 expression and TME-related biological processes in 33 tumors is presented in Figure 10(e). Except in UCS, CESC, HNSC, and DLBC, BOP1 expression is positively linked with DNA damage repair-related pathways in 29 malignancies. Furthermore, in OV, KICH, ACC, PCPG, BLCA, and KIRP, BOP1 expression is positively associated with TMEscoreA, but in UCS, LGG, PRAD, HNSC, SKCM, SRAC, THCA, and LUSC, it is negatively correlated with TMEscoreA. Moreover, it suggested that high BOP1 expression is positively related to TMEscoreB in patients with OV, KICH, ACC, PCPG, GBM, and THCA, but that BOP1 expression and TMEscoreB are negatively related in LGG, CESC, PRAD, HNSC, THYM, SKCM, SARC, TGCT, LUSC, ESCA, LIHC, UCEC, BRCA, LUAD, PAAD, STAD, BLCA, and COAD.

Subsequently, we analyzed the correlation of BOP1 expression with the infiltration of 24 immune-related cells in 33 cancers. In most cancers, BOP1 expression was shown to be strongly related to the levels of immune cell infiltration (Figure 10(f)). Among them, BOP1 expression was found to be positively related to the levels of infiltrating natural regulatory (nTreg) cell (except in THCA and THYM), Th1 cell (except in LGG, SKCM, and THCA), neutrophil cell (except in THYM), and monocyte cell (except in TGCT, THCA, and THYM) and negatively related to the levels of infiltrating central memory T (Tcm) cell, NK cell (except in KIRC), CD4-T cell (except in GBM and THCA), mucosal associated invariant T (MAIT) cell (except in THCA and MESO), and follicular helper T (Tfh) cell (except in KICH and THYM).

### 3.6. Correlation of BOP1 Expression with Immune Checkpoints, MSI, and TMB in 33 Tumors

**Figure 11:**
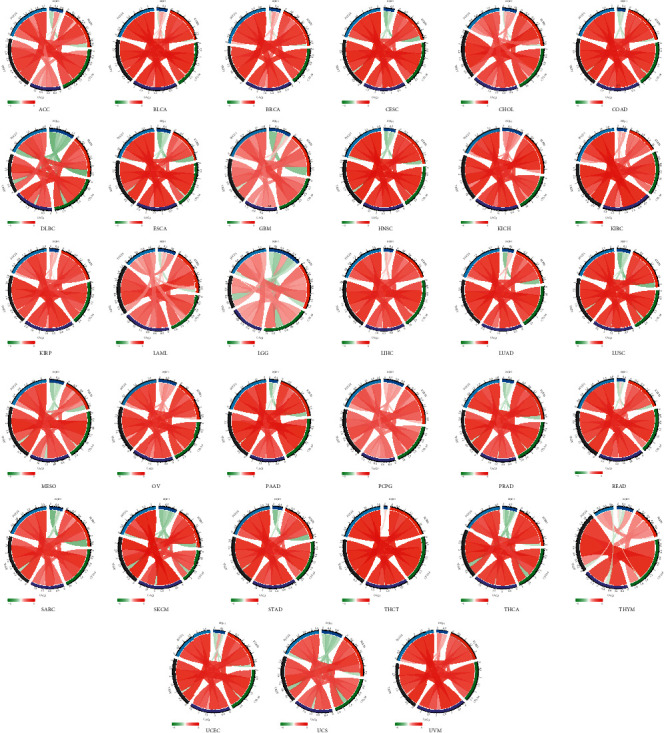
Correlation of BOP1 expression and immune checkpoints in pan-cancer.

**Figure 12:**
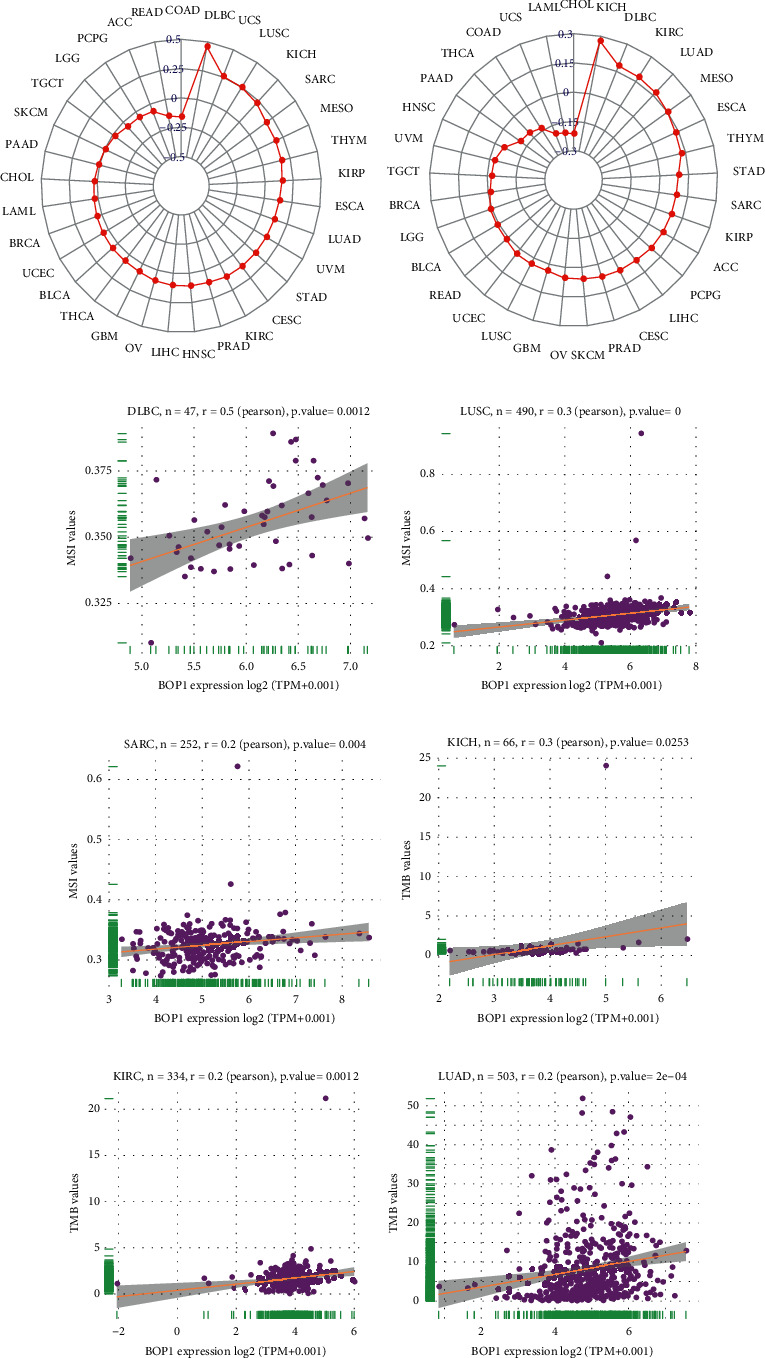
Correlation of BOP1 expression and MSI and TMB in pan-cancer. (a) The relationship between BOP1 expression and MSI in 33 tumors. (b) The relationship between BOP1 expression and TMB in 33 tumors. (c–e) The relevance between BOP1 expression and MSI in DLBC, LUSC, and SARC. (f–h) The relevance between BOP1 expression and TMB in KICH, KIRC, and LUAD.

Immune checkpoints are closely related to tumor immunotherapy. Here, we analyzed the relationship between five major immune checkpoints (PDCD1, KLRB1, CTLA4, LAG3, and TIGIT) and BOP1 expression. We found that the BOP1 expression level is significantly associated with the expression of these immune checkpoint genes in the majority of malignancies (Figure 11); it suggests that BOP1 might be used as a potential biomarker to guide tumor immunotherapy. Tumor immunotherapy relies heavily on MSI status. Considering that MSI is frequently caused by functional defects of the mismatch repair system, and that BOP1 expression is related to mismatch repair, we analyzed the relationship between BOP1 level and MSI in 33 cancer types. BOP1 expression was shown to be favorably correlated with MSI in LUSC, DLBC, LUAD, SARC, KIRP, STAD, PRAD, HNSC, KIRC, CESC, and LIHC but negatively in COAD. Besides, we also analyzed the TMB of each sample, which is also closely associated with cancer prognosis and response to immunotherapy. It has been suggested that BOP1 positively linked to TMB in LUAD, KIRC, THCA, COAD, and KICH and negatively associated with TMB in LAML (Figure 12).

### 3.7. Expression and Functional Verification of BOP1 in Lung Cancer Cells

**Figure 13:**
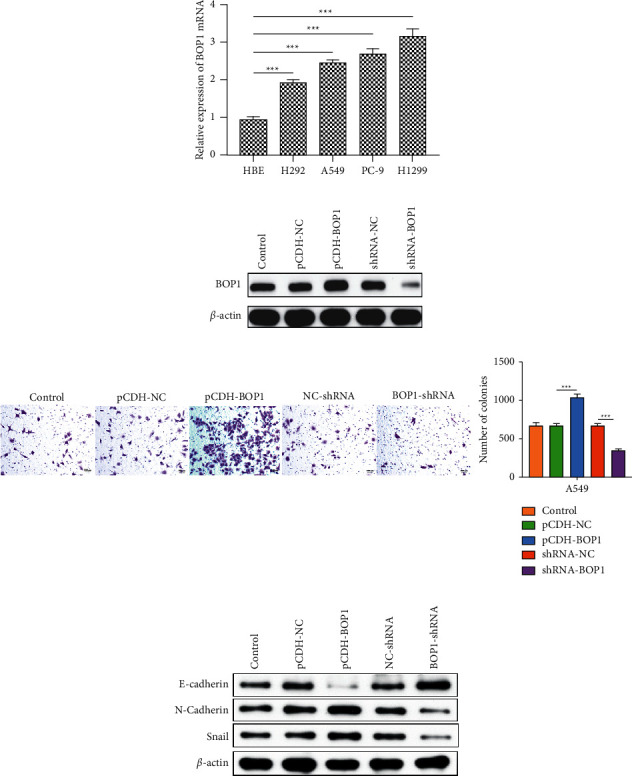
The expression and potential function of BOP1 in lung cancer cells. (a) The expression of BOP1 mRNA in HBE, H292, A549, PC-9, and H1299 cell. (b) The expression of BOP1 in A549 cells stably transfected with BOP1-shRNA or pCDH-BOP1. (c) The migration ability of BOP1-overexpressed or BOP1-knockdown A549 cells. (d) The protein expression of EMT-related genes (snail, N-cadherin, and E-cadherin) in BOP1-overexpressed or BOP1-knockdown A549 cells. ^*∗∗∗*^*P* < 0.001.

We detected the expression levels of BOP1 in lung cancer cell lines H292, H1299, A549, PC-9, and human bronchial epithelioid cell line HBE, and the results showed that BOP1 was significantly augmented in lung cancer cells compared with HBE cell (Figure 13(a)). Cell migration was greatly aided by increased BOP1 expression, whereas its downregulation displayed the opposite impact (Figures 13(b) and 13(c)). Considering that our previous analysis indicated that BOP1 expression was associated with EMT, we explored the relationship between BOP1 and EMT-related genes. As shown in Figure 13(d), BOP1 silencing diminished snail and N-cadherin expressions but upregulating E-cadherin. In the BOP1 overexpression cells, the opposite phenomenon was observed.

## 4. Discussion

BOP1, a member of the WD40 protein family with four WD repeat motifs, including 732 amino acids, is highly conserved in eukaryotes and has been identified as a vital regulator in 60S ribosome biogenesis and ribosomal RNA processing [16, 17]. The BOP1 protein is an important component of the PeBoW complex, which is required for large ribosomal subunits maturation and cell proliferation [18, 19]. BOP1 has been reported to upregulate in several cancers in recent years, and it is been linked to tumor metastasis, migration, and poor prognosis [8, 9]. However, the expression, clinical significance, and biological effect of BOP1 in the vast majority of tumors remain largely unknown.

In the current study, we executed a comprehensive investigation of BOP1 expression, prognostic value, and potential function in 33 tumors. We found that BOP1 mRNA was upregulated in 25 types of cancer and downregulated in LAML and THCA samples. The results were consistent with previous studies in gastric cancer [8], colorectal cancer [10, 20], prostate cancer [9], triple-negative breast cancer [21], and hepatocellular carcinoma [13]. These findings suggested that BOP1 is likely to operate as an oncogene in most tumors. Previous studies have also clarified that that BOP1 serves as an oncogene in tumorigenesis and progression of certain carcinomas [8, 12, 22]. Further investigation revealed that BOP1 exhibits CNV amplification and hypomethylation of its promoter region in the majority of tumors, which might be a contributing factor to BOP1 high expression. Furthermore, a recent study reported that the expression of BOP1 protein was downregulated in melanoma patients, and that BOP1 loss was related to BRAF kinase resistance [23].

We also investigated whether BOP1 expression is associated with prognosis of cancer patients. The results showed that the upregulation of BOP1 is a risk factor in most cancers and is related to DFI, DSS, PFI, and OS. The Kaplan–Meier survival analysis showed that increased BOP1 expression was correlated with poor OS in nine tumors. Similarly, it was previously reported that BOP1 expression is related to shorter survival period in patients with prostate carcinoma [9], triple-negative breast cancer [21], gastric cancer [8], and hepatocellular carcinoma [24], and BOP1 promotes the development of these cancers. Moreover, BOP1 expression might be used as a potential prognostic marker in patients with tumor metastases [9, 21]. By contrast, the opposite was found with a relationship between BOP1 expression and prognosis in LGG patients. Our results also indicated that the BOP1 promoter methylation level was positively related to survival time in tumor patients. These findings clearly indicated that BOP1 may be used as a molecular marker to predict the prognosis of different cancers.

Tumor microenvironment plays important roles in the occurrence, progression, and metastasis of cancers [25–27]. Our results showed that BOP1 expression is correlated with tumor microenvironment involving immune regulation, DNA damage repair, and EMT. Of these, a positive connection was observed between BOP1 expression and DNA damage repair-related pathways in 29 cancers. DNA repair deficiency can lead to the buildup of DNA damage in the genome, which is an important cause of cancer [28, 29]. These findings showed that BOP1 affects DNA damage repair and hence plays a key role in carcinogenesis. Immune cell infiltration is closely related to clinical outcomes of cancer patients [30, 31].We also evaluated how BOP1 expression correlated with the infiltration of 24 immune-related cells in 33 malignancies. We found that BOP1 was positively linked with the nTreg cell levels and negatively associated with the infiltrating Tcm cell, NK cell, and Tfh cell. Previous studies have shown that Treg has an immunosuppressive effect and promotes immune escape of tumor cells, whereas Tcm cell, NK cell, and Tfh cell have superior antitumor properties [32–34]. It is suggested that BOP1 interconnected and interacted with immune cells to promote cancer progression. In addition, previous studies have shown that MSI is also commonly caused by functional defects in the DNA mismatch repair system [35, 36], and BOP1 expression is related to DNA mismatch repair, so we evaluated the relevancy between BOP1 expression and MSI. Then, a positive correlation was identified between BOP1 expression and MSI in 12 tumors. Similarly, TMB is also linked to a longer post-treatment survival time in patients with tumors, implying that it might act as a molecular indicator to instruct the clinical decision-making of tumor immunotherapy, targeted therapy, and adjuvant chemotherapy [37, 38]. Here, we found that BOP1 was linked with TMB in 5 tumors. Overall, our findings show that BOP1 expression is linked to the tumor microenvironment, immune cell infiltration, MSI, and TMB; impacts patient prognosis; and gives new insights into immunotherapy and immunosuppressive drug development.

Finally, the RT-qPCR, WB, and transwell assay were carried out to verify related findings in lung cancer cell lines. The results indicated that BOP1 was significantly augmented in lung cancer cells compared with HBE cell. The transwell assay showed that the enhanced expression of BOP1 significantly facilitated cell migration, but its downregulation had the opposite impact. These data support our prior findings that BOP1 is overexpressed in malignancies and functions as an oncogene. Furthermore, silencing BOP1 diminished snail and N-cadherin expression but increased E-cadherin expression, confirming the link between BOP1 expression and EMT.

In conclusion, we performed the first pan-cancer analysis of BOP1, which indicated a substantial difference in BOP1 expression between normal and tumor tissues, as well as a link between BOP1 expression and patient prognosis. Our research results show that BOP1 can be used as a promising molecular prognostic biomarker for various tumors. The expression of BOP1 in various malignancies will result in varied clinical outcomes, necessitating a large number of verification experiments to investigate the particular biological role of BOP1 in each cancer. In a variety of cancers, BOP1 expression has also been related to the tumor microenvironment, immune cell infiltration, MSI, and TMB, albeit its effect on tumor immunity varies. These results contribute to clarify the role of BOP1 in tumorigenesis and progression, as well as new insights into the future development of more personalized, precise tumor immune-targeting therapies.

## Data Availability

Publicly available datasets were analyzed in the present work. These data can be found in the UCSC XENA website: https://xenabrowser.net/datapages/.

## Supplementary Materials

Supplementary Materials Table S1: correlation of BOP1 expression with BOP1 promoter methylation.
