# Supplementary material for: BOP1 Used as a Novel Prognostic Marker and Correlated with Tumor Microenvironment in Pan-Cancer
Source: J Oncol. 2021 Sep 22;2021:3603030. doi: 10.1155/2021/3603030 (PMC8481050; doi:10.1155/2021/3603030)
Supplement: Supplementary Materials — Table S1: correlation of BOP1 expression with BOP1 promoter methylation. [file 3603030.f1.docx]

| **Correlation** | **P-value** | **Cancer** | **Result** |
| --- | --- | --- | --- |
| -0.56941214 | 4.35E-08 | UVM | negtive |
| -0.487660758 | 0.000119289 | UCS | negtive |
| -0.47590953 | 3.25E-16 | SARC | negtive |
| -0.472672337 | 5.67E-29 | PRAD | negtive |
| -0.457631565 | 8.70E-42 | BRCA | negtive |
| -0.405605742 | 1.34E-08 | ESCA | negtive |
| -0.389345188 | 9.08E-15 | LUSC | negtive |
| -0.387891859 | 2.98E-18 | SKCM | negtive |
| -0.362137971 | 3.46E-17 | THCA | negtive |
| -0.348980963 | 2.44E-16 | HNSC | negtive |
| -0.347199645 | 0.016798234 | DLBC | negtive |
| -0.336875088 | 2.94E-12 | BLCA | negtive |
| -0.326788167 | 0.002006443 | MESO | negtive |
| -0.325581837 | 0.003859648 | ACC | negtive |
| -0.306865784 | 2.52E-05 | PCPG | negtive |
| -0.11898 | 0.034214 | KIRC | negtive |
| 0.134327543 | 0.026463663 | KIRP | positive |

**Table S1 Correlation of BOP1 expression with BOP1 promoter methylation**
